# Identification of three anatomical patterns of the spinal accessory nerve in the neck by neurophysiological mapping

**DOI:** 10.2478/raon-2013-0069

**Published:** 2014-11-05

**Authors:** Bostjan Lanisnik, Miha Zargi, Zoran Rodi

**Affiliations:** 1 Department of Otorhinolaryngology, Cervical and Maxillofacial Surgery, University Medical Center Maribor, Slovenia; 2 Department of Otorhinolaryngology and Cervicofacial Surgery, University Medical Center Ljubljana, Slovenia; 3 Institute of Clinical Neurophysiology, University Medical Center Ljubljana, Slovenia

**Keywords:** spinal accessory nerve, nerve mapping, neck dissection, anatomy, shoulder disability

## Abstract

**Background:**

In spite of preservation of the accessory nerve there is still considerable proportion of patients with partial nerve damage during modified radical neck dissection (MRND).

**Methods:**

The nerve was identified during the surgery and its branches for the trapezius muscle mapped with nerve monitor.

**Results:**

The accessory nerve was mapped during 74 hemineck dissections and three patterns were identified. In type 1 nerve exits at the posterior end of the sternocleidomastoid muscle (SCm) and then it enters the level V (66%). In type 2 the nerve for trapezius muscle branches off before entering the SCm (22%). In type 3 the nerve exits at the posterior part of the SCm and it joins to the cervical plexus (12%). The nerve than exits this junction more medially as a single trapezius branch.

**Conclusions:**

The description of three anatomical patterns in level II and V could help preserving the trapezius branch during MRND.

## Introduction

Accessory nerve provides motor supply to sterno-cleidomastoid and trapezius muscle. If the nerve is damaged during the surgery this results in a trapezius muscle weakness, atrophy and shoulder syndrome. Cranial and middle portions of trapezius muscle are innervated with accessory nerve proper, while caudal portions of the muscle thought to be innervated with fibers originating from C3 and C4 cervical plexus, but some authors believe that contributions from the cervical plexus are purely proprioceptive.^1^,^2^ Anatomy of the accessory nerve in the anterior and posterior triangle of the neck has been comprehensively described in the literature.^3^ Even though today the nerve is routinely preserved during neck dissection whenever possible, some authors demonstrate that functional outcomes after the modified radical neck dissection are worse compared to selective neck dissection.^4^,^5^ This is attributed to the dissection of the level V and resection of the cervical roots.^6^,^7^

In this study we mapped the accessory nerve during modified radical neck dissection type 3, using electrophysiological techniques with the goal to describe surgical anatomy and nerve variations which might be helpful in improving functional outcomes after surgery.

## Patients and methods

Forty patients were studied during the neck dissection for head and neck cancer from January 2012 to January 2013. The aim of the study was to map the course of the accessory nerve, with special consideration to the innervation of the trapezius muscle. The study was designed as a prospective cohort study of patients undergoing first treatment for head and neck cancer without previous surgery or radiation therapy. All patients had N0 or N+ neck that warranted modified radical neck dissection type 3 on one or both sides with preservation of the cervical roots. The study was approved by the Committee for Medical Ethics of the Republic of Slovenia.

Modified radical neck dissection type 3 (MRND) was performed with anterior to posterior dissection technique with preservation of the cervical plexus. The dissection started with elevation of the apron flap. Superficial layers of the deep cervical fascia were incised over the sternocleidomastoid muscle and retracted medially. The muscle was dissected of the fascia to the entrance of the accessory nerve into the muscle. This anatomical point is predictable and is easily identified. The dissection of the accessory nerve continued in the cranial direction, where all branches were preserved. After that, we identified the branch for the trapezius muscle at the posterior margin of the sternocleidomastoid by dissecting the fascia from the muscle with the help of the nerve monitor. Dissection in level V and IIb continued bellow the cervical plexus and accessory nerve, carefully preserving all branches and removing all fatty tissue and fascia. The dissection ended on the alar layer of the deep fascia. Integrity of the accessory nerve was tested continuously during the procedure.

**FIGURE 1.**
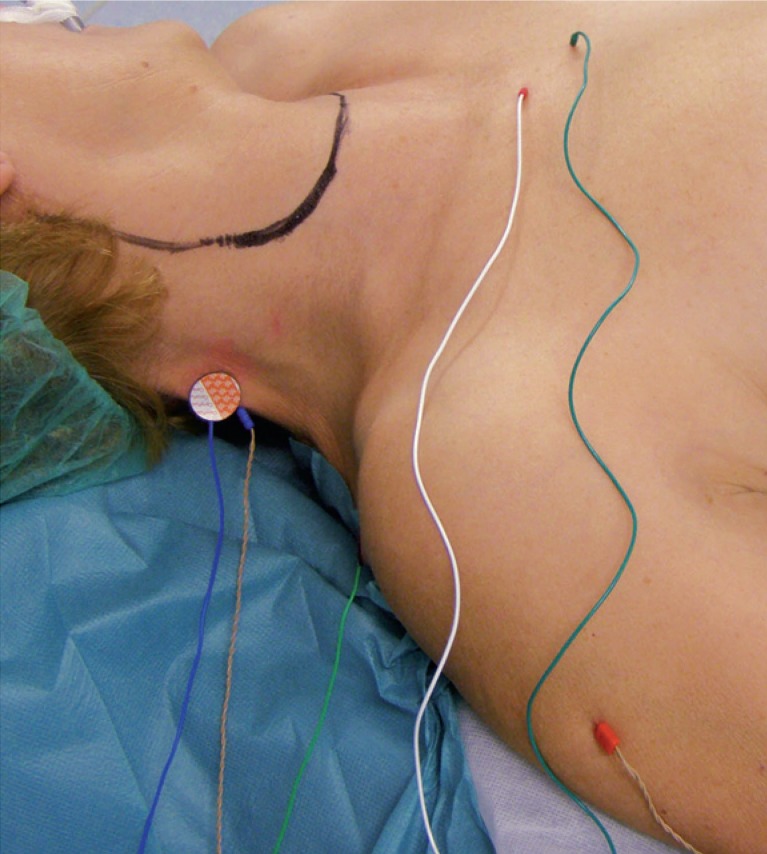
Placement of the electrodes in the patient before surgery. Surface electrodes over the trapezius are seen as well as needle electrodes in the trapezius and deltoid muscles. Reference and ground electrodes are placed over the *jugulum*.

The mapping of the accessory nerve was performed using an intraoperative nerve monitoring device NIM 2.0 (Medtronic Navigation Inc., USA) with bipolar needle and surface electrodes placed in/over the trapezius muscle (registering electrode over the biggest muscle mass and negative electrode over the acromion, Figure 1). A needle electrode was also placed in the deltoid muscle to detect a possible contamination from the stimulation of the brachial plexusSurface electrodes record compound muscle action potentials (CMAP) from a much wider area compared to needle electrodes thus giving better estimation of the number of excitable motor axons innervating the given muscle. On the other hand, bifocally placed needle electrodes record the CMAP only between the needle tips in a localized area of the muscle thus minimizing the risk of recording the action potential from surrounding muscles but at the same time detecting only the smaller fraction of motor axons. Only stimulations with responses recorded on both needle and surface electrodes placed in/over trapezius muscle, and at the same time showing no response in deltoid muscle, were considered to originate from the accessory nerve or its branches.

The nerve was mapped (stimulated) using a hook stimulating electrode that was used to lift up the nerve and prevent the stimulus from spreading in the surrounding tissue. Stimulus current was gradually increased to achieve a supramaximal stimulation. We also stimulated the cervical roots as medially as possible to exclude potential concurrent stimulation of the accessory nerve or its branches. The nerve and roots producing maximal and identical response in the trapezius muscle were followed. The anatomical course of the accessory nerve was recorded.

## Results

Mapping of the accessory nerve during 74 modified neck dissections, type 3, was performed in 40 patients. Eighteen patients were treated for oral cavity cancer, 8 for laryngeal cancer, 4 for hypopharyngeal carcinoma, 8 for oropharyngeal cancer, 1 for skin cancer and 1 patient for carcinoma of unknown primary. Mapping of the accessory nerve in 74 dissections resulted in identification of three predictable and recurring anatomical patterns of the accessory nerve.

**FIGURE 2.**
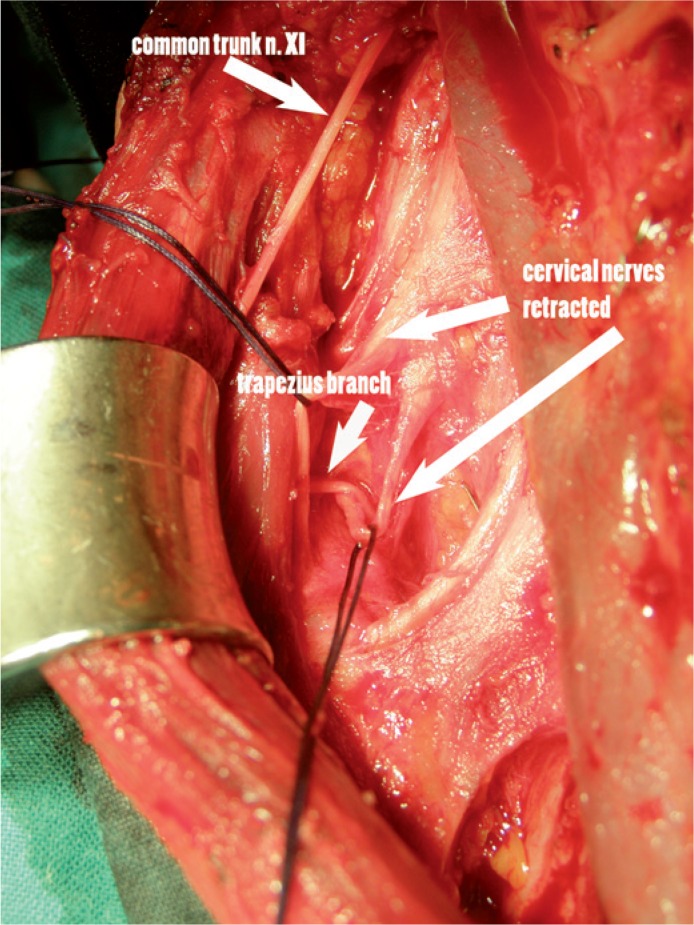
In type 1 pattern, a trapezius branch exits at the posterior margin of the sternocleidomastoid muscle, deep to the cervical nerves, and continues to enter level V. Cervical roots are displaced with suture loops for better visualization of the trapezius branch. This pattern was present in 69% of the patients.

Type 1 pattern of the accessory nerve was the most common and was found in 49 out of 74 heminecks (66%). In this pattern, the nerve coursed through the sternocleidomastoid muscle, where it divided into the sternocleidomastoid branch and trapezius branch. The latter exited the muscle at its posterior edge and is readily identified in the posterior part of region V and deep to the plane of the cervical roots (Figure 2).

**FIGURE 3.**
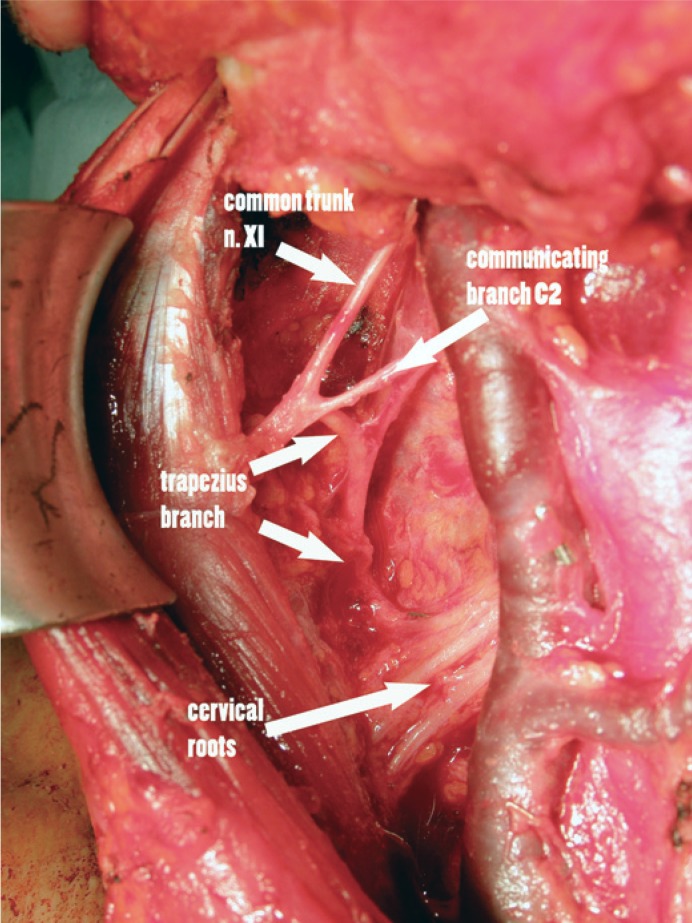
In type 2 pattern, a trapezius branch exits the main trunk at level IIa and continues superficial to the cervical nerves in level IIa and enters level V. In this figure, we can see that it is essential to identify this branch early during the dissection in level IIa. This pattern was found in 20% of patients.

Type 2 pattern was identified in 16 out of 74 heminecks (22%). In this case the branch for the trapezius muscle branched off the common trunk before entering the sternocleidomastoid muscle. The course of this branch is superficial to the cervical roots at least to the level of C4, where it coursed deep and entered region V and the trapezius muscle (Figure 3).

**FIGURE 4.**
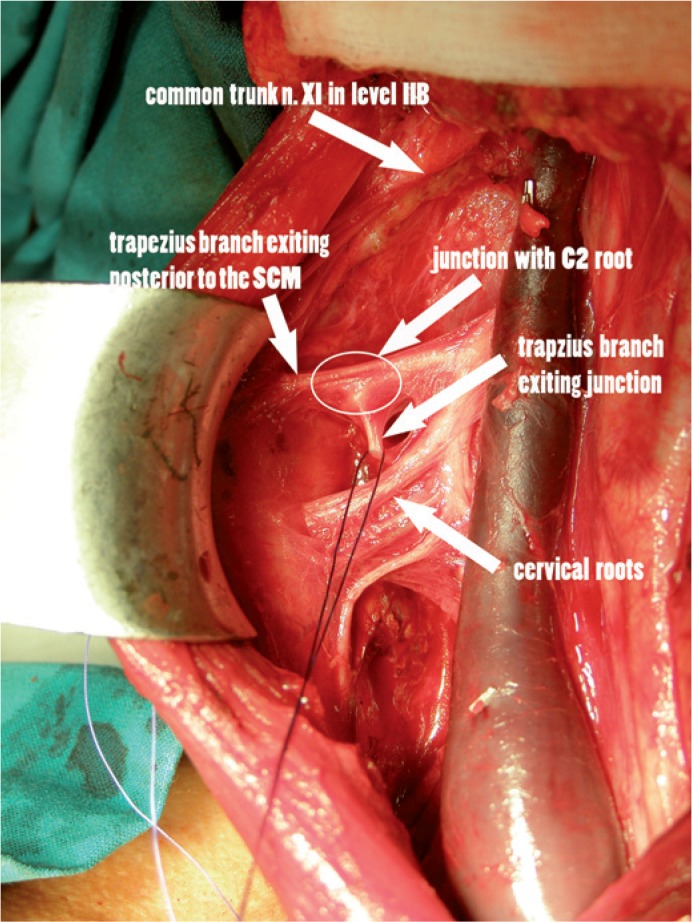
Type 3 pattern is more complicated. The trapezius branch is seen exiting at the posterior margin of the sternocleidomastoid muscle and joining with cervical nerves. From this junction, a single branch exits more medially and enters level V (branch is looped with suture). If this type is not recognized, the branch that enters level V might be mistaken for a cervical nerve and cut. This pattern was present in 11% of all patients. SCM-sternocleidomastoid muscle

Type 3 is the least common and was found in 9 out of 74 heminecks (12%). It is the most complicated pattern, where the trapezius branch exited at the posterior end of the sternocleidomastoid muscle and joined to the cervical roots at level C3 and/or C4. The trapezius branch then exited from this junction more medially and then it turned deep to the level V (Figure 4).

Thirty-four patients had bilateral modified radical neck dissection. In twenty-seven patients (79%) the same type was found on both sides of the neck. Type 1 was found in 58.8% (20 out of 34) patients, type 2 14.7% (5 out of 34) patients and type 3 in 5.9% (2 out of 34) patients (Table 1). In other 7 patients the nerve types were present in different combinations: type 1- type 2 in 2 patients (5.9%), type 2-type 3 in 2 patients (5.9%) and type 1-type 3 in 3 patients (8.8%).

In all 40 patients and 74 dissections, we identified a single branch for the trapezius muscle entering region V where it divided into branches for different parts of the muscle.

**FIGURE 5.**
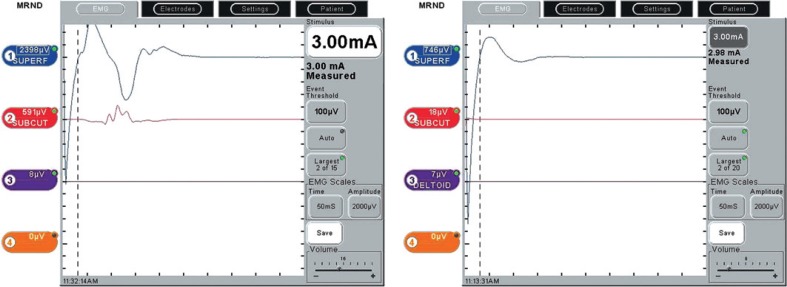
The recording of the compound action potential (CMAP) during mapping can be seen on the left side in a patient with type 3 branching. Channel 1 records superficial electrodes while Chanel 2 records from the subcutaneous electrodes. Channel 3 records the CMAP of the deltoid muscle. The left image represent the stimulation of the accessory nerve branches, while the right represents recording while stimulating the cervical branches of C4 where only stimulus artifact and not the CMAP is recorded. Similar results were obtained if we stimulated the C2 communicating branch.

We couldn’t identify the motor response in the trapezius muscle when we stimulated different parts of the cervical roots C2, C3 or C4. If higher currents were used, we could identify the electrical stimulus artifacts on the superficial electrodes when stimulating cervical roots or different tissues of the neck (Figure 5). We identified C2 communication branch in all 74 dissections, but its thickness varied considerably. This branch always led to the trapezius branch and proved to be a reliable marker during the dissection.

## Discussion

The anatomical course of the accessory nerve in the neck is described in numerous articles. The main identification point of the nerve is in the posterior triangle, behind the posterior edge of the sternocleidomastoid muscle at Erb’s point, which is defined by the exit of the greater auricular nerve from behind the sternocleidomastoid muscle. The accessory nerve can also be identified at the entry point into the sternocleidomastoid muscle. This is the perspective of the nerve identification during modified radical neck dissection. The nerve passes lateral to the jugular vein in the majority of the cases, while medial passage is rare, but the incidence varies in the literature.^3^,^8^

Even though the accessory nerve is preserved in its superior part during a modified radical neck dissection, significant postoperative shoulder disability can still be observed. This is attributed to the resection of the cervical branches and lymph node dissection in levels IIb and V that leads to damage to branches to the trapezius muscle. Those findings led Kierner *et al.* to conclude that traditional anatomical concepts of the topography of the accessory nerve are not correct. They described a small cranial branch that takes off in the posterior triangle from the main trunk of the accessory nerve and enters into the descending part of the trapezius muscle.^9^,^10^

Lee *et al.* studied the anatomy of the accessory nerve at level IIb. They found that the nerve passed ventrally to the internal jugular vein in 39.8% of the cases, dorsally in 57.4% and through it in 2.8% of the cases. They also described that in 45.9% of the cases, the nerve sent branches to the sternocleidomastoid muscle without penetrating it, whereas in 54.1% of the cases the nerve passed through the muscle.^11^

Shiozaki *et al*. performed a detailed cadaveric anatomical study with special emphasis on different types of sternocleidomastoid innervation. They described three types: type A is a non-penetrating type; in type B, the nerve partially penetrates the sternocleidomastoid muscle; and in type C, it completely penetrates the muscle. They also described 5 different types of trapezius branches based on the number of branches that innervate the anterior margin of the muscle.^12^

The contribution of the cervical roots to the innervation of the trapezius is still controversial. Current understanding that the trapezius has mixed innervation is based on electromyographical studies of Haas and Solberg from 1962, who stated that the innervation to the trapezius was received from cervical and thoracic branches as well as from spinal accessory nerve.^13^ Kierner *et al.* pointed out that their methodology might be questionable, because of the spread of the stimulating current in the posterior triangle, if proper care during the stimulation is not taken. This can lead to false positive results.^10^

After searching the National Library of Medicine database on line (PubMed) we could identify three studies in the last 30 years, where electrophysiological technique was used to address this question. Soo *et al.* used needle detector electrodes in upper, middle and lower part trapezius muscle. They compared the size and pattern of the CMAP if accessory nerve and cervical plexus was stimulated and identified three patterns of motor action potentials. The authors concluded that the trapezius muscle innervation from the cervical plexus is present, but unpredictable and that most important motor input came from accessory nerve.^2^

Pu *et al.* also used electroneuronography to identify the contribution of the cervical plexus to the innervation of the trapezius.^14^ They compared CMAP from the accessory nerve, C2, C3 and C4 contributing branches, before and after sectioning the nerve during surgery. They concluded that main motor supply is from the spinal accessory nerve with variable contributions from C2-C4 branches. They also used histochemical staining for acetylcholine esterase activity and found that 0/19 C2 branches, 1/13 C3 branches and 1/14 C4 branches contained motor axons.

Kierner *et al.* used electromyography to identify a small cranial branch that innervates the descending part of the trapezius muscle. This branch takes off in the posterior triangle of the neck medial to the trapezius muscle. The authors could not demonstrate any clinically relevant contribution from the cervical branches to the motor innervation of the trapezius muscle.^10^ They described that in about a third of the patients the nerve ran dorsally to the sternocleidomastoid muscle and not through it.

The results of our work, where we mapped the nerve using electrophysiological technique similar to Kierner’s *et al.* confirmed some of his observations. We used surface electrodes for detection of the muscle response and we didn’t measure the response in different parts of the muscle. The rationale for this decision was the fact that we were mapping the nerve before it branched in the posterior triangle. The branching in the posterior triangle is of great importance in posterolateral dissection. We could identify three major patterns of the accessory nerve in its neck course. In type 1, branches for the trapezius exit the sternocleidomastoid muscle in the area of the Erb point, deep to the cervical plexus and take a course in the posterior triangle as it is usually described in the anatomical dissections.^3^,^8^,^15^ Type 2 pattern is present when trapezius branches exit the nerve before entering the sterno-cleidomastoid muscle. This means that the first part of the course is anterior (above the plane) to the cervical plexus and it enters the posterior triangle at various levels, but always bellow C2. This pattern corresponds to the Kierner’s *et al.* observation in one third of the patients where the accessory nerve passed through the sternocleidomastoid muscle (22% in our series).^9^,^10^

The most interesting pattern identified is type 3 where the nerve exits at the posterior surface of the sternocleidomastoid muscle and it joins with the cervical plexus at the C3–4 level. From there, a single branch for the trapezius muscle exits medial to this junction. This medial positioned branch can be confused for the cervical root and cut during the dissection. This type could also explain the confusion regarding the contribution of the cervical nerves in innervation of the trapezius. When approaching the nerve from a posterior to anterior direction, it is almost impossible to identify the branch that exits from the cervical plexus and innervates the trapezius muscle.

During the modified radical neck dissection the surgeon encounters the accessory nerve from different perspectives. In the beginning of the procedure the nerve is identified at level II before entering into the sternocleidomastoid. If a type 2 division is present, the trapezius branch takes off before entering the sternocleidomastoid muscle (Figure 3). Therefore the trapezius branch is encountered early during the procedure and must be preserved. In type 1, the nerve can be safely identified behind the posterior edge of the sternocleidomastoid muscle, before passing through level V. Identification of the C2 branch also leads to the trapezius branch and is a safe landmark (Figure 2). In type 3, it is of paramount importance to preserve cervical branches since the branch after exiting from the sternocleidomastoid muscle intermixes with cervical nerves at levels C3 to C4. From there, a single branch exits and enters level V, which can than be safely followed and dissected (Figure 4).

## Conclusions

The description of anatomical variations of the accessory nerve in level IIb and V is of clinical importance during neck dissection. Even though the accessory nerve is preserved during modified radical neck dissection, there is still considerable morbidity of the shoulder girdle. We believe that at least some damage to the nerve is inflicted during neck dissection, because the anatomy of the nerve in levels IIb and V is not sufficiently understood by the surgical community.

The description of three different types of trapezius branching patterns might help in its identification and decrease morbidity during neck dissection.

## Figures and Tables

**TABLE 1.** The table shows frequencies of nerve type combinations in the left and right side of the neck in patients with bilateral modified radical neck dissection

**Nerve type combination**	**No. of patients (%)**
Type 1 – Type 1	20 (58.8 %)
Type 2- Type 2	5 (14.7 %)
Type 3- Type 3	2 (5.9 %)
Type 1- Type 3	3 (8.8 %)
Type 1- Type 2	2 (5.9 %)
Type 2- Type 3	2 (5.9 %)
